# Aberrant Salience Network Functional Connectivity in Resting-State and Fear-Related Autobiographical Memory Recall in Female Adolescents with Borderline Personality Disorder

**DOI:** 10.3390/brainsci15111146

**Published:** 2025-10-25

**Authors:** Elena De Rossi, Chiara Di Maggio, Claudio Imperatori, Marilina Covuccia, Giuseppe A. Carbone, Arianna Terrinoni, Chiara Massullo, Vincenzo Guidetti, Mario Brinciotti, Giulia Biscione, Benedetto Farina

**Affiliations:** 1 Experimental and Applied Psychology Laboratory, Department of Health and Life Sciences, European University of Rome, 00163 Rome, Italy; elena.derossi@unier.it (E.D.R.); claudio.imperatori@unier.it (C.I.); 2 Child and Adolescent Neuropsychiatry Unit, Department of Human Neuroscience, Sapienza University of Rome, 00185 Rome, Italy; c.dimaggio.npi@gmail.com (C.D.M.); marilina.covuccia@uniroma1.it (M.C.); a.terrinoni@policlinicoumberto1.it (A.T.); vincenzo.guidetti@uniroma1.it (V.G.); mario.brinciotti@gmail.com (M.B.); giuliabiscione00@gmail.com (G.B.); 3 Child and Adolescent Neuropsychiatry Unit, Department of Mental Health, ASL Roma 4, 00063 Campagnano di Roma, Italy; 4 Child and Adolescent Neuropsychiatry Unit, Department of Mental Health, ASL Roma 5, 00034 Colleferro, Italy; 5 Faculty of Psychology and Educational Sciences, University of Geneva, 1205 Geneva, Switzerland; giuseppealessio.carbone@unito.it; 6 Department of Education, Roma Tre University, 00154 Rome, Italy; chiara_massullo@yahoo.it; 7 Child and Adolescent Neuropsychiatry Unit, Department of Mental Health, ASL Roma 5, 00019 Tivoli, Italy

**Keywords:** borderline personality disorder, inpatient adolescents, salience network, EEG functional connectivity, autobiographical memories

## Abstract

**Objectives.** Identity disturbance and instability in Borderline Personality Disorder (BPD) are associated with impairments in the integration of emotional autobiographical memory (EAM). At the neurophysiological level, it has been suggested that EAM dysfunction may be linked with functional connectivity (FC) alterations of the salience network (SN). Despite this, evidence in adolescents with BPD remains scarce, especially under task-related conditions. Therefore, we investigated SN electroencephalography (EEG) FC in adolescents with BPD during the resting-state condition (RS) and during two EAM tasks (i.e., happiness- and fear-related). **Methods.** A total of 24 female adolescents with BPD and 15 healthy controls underwent RS and task-related EEG recording. All participants were also assessed for BPD and related clinical dimensions. EEG FC analyses in the SN were performed using exact Low-Resolution Brain Electromagnetic Tomography (eLORETA) software. **Results.** Compared to controls, BPD patients exhibited reduced theta SN connectivity during RS. This hypo-connectivity pattern was positively correlated with all BPD-related dimensions (i.e., emotional dysregulation, impulsiveness, dissociative symptoms, and childhood trauma). Furthermore, compared to the RS, during the listening of fear-related memories, BPD patients showed an increase in delta SN connectivity. This hyper-connectivity pattern was negatively correlated with the self-reported vividness of recall. **Conclusions.** While decreased SN theta connectivity may be a common neural marker of traumatic disintegration, increased SN delta connectivity may indicate a neural correlate of suppression/avoidance of negative memories.

## 1. Introduction

Borderline Personality Disorder (BPD) is a severe psychiatric condition considered a significant public-health concern [1,2,3,4]. Indeed, it has been associated with higher rates of self-injury and suicidal behavior [5], poorer outcomes in the transition to adulthood [6], and long-term functional deficits [7]. Furthermore, as BPD also typically emerges in early adolescence and peaks between approximately 14 and 17 years of age, there is general agreement that its diagnosis requires early recognition and treatment [8].

In the last decade, research on the developmental signatures of the disorder has significantly increased, shading light on several psychopathological processes that may underlie the onset and maintenance of the symptoms [9]. Indeed, BPD is considered a complex clinical disorder arising from the interaction between genetic factors and adverse childhood experiences [10].

At the neurophysiological level, strong evidence has been found on the altered connectivity between frontal and limbic structures that has been associated with higher emotional dysregulation (e.g., [11,12]). However, other crucial symptoms of the disorder are largely unexplored, such as the complex configuration of symptoms related to identity disturbance and instability [13,14].

It has been suggested that the hampered construction of a coherent self-narrative and a fragmented sense of self in BPD may be caused by autobiographical memory disturbances [15], and the interest in the neural underpinnings underlying this process is considerably growing. Autobiographical memory (i.e., the memory system storing time-located experiences that are meaningful to the self; [16]) is thought to develop through early childhood experiences in the relationship with the caregivers. If properly developed, it contributes to orienting attention, emotional responses, and behaviors in daily life [17] and to consolidating interpersonal relationships through the sharing of life experiences with others [18]. Autobiographical memories often involve powerful emotional content and are more arousing than neutral memories, more easily encoded in memory (e.g., [19]), more easily retrieved (e.g., [20]), and more effective in guiding future approach/avoidance behaviors (e.g., [21]).

At the neurophysiological level, emotional autobiographical memory is a high-order cognitive function requiring high integration of neural networks [22]. In particular, it has been suggested that autobiographical memory dysfunction, especially for negative memories, may be linked with alteration of brain connectivity of the salience network (SN) in psychiatric populations [23]. The SN is mainly anchored in the anterior insula and the dorsal anterior cingulate cortex (ACC; [24]) and is involved in the detection of relevant internal and external stimuli and in the coordination of an appropriate behavior [25]. Its aberrant functioning is highly associated with psychopathology [26] and it has been shown to have a mediating role in the relationship between childhood trauma and mental health in adolescence [27,28]. Furthermore, several studies conducted in adult patients with BDP found aberrant resting state (RS) connectivity in the SN [29,30,31,32,33].

Despite the crucial role of identity disturbance and instability in BPD onset and maintenance [14,34] and the alterations in the underlying cognitive process of emotional autobiographical memory [15], few on-task studies have assessed alterations in the brain networks (e.g., SN) that could underlie this symptom dimension. Furthermore, in recent years, neuroscience studies have consistently improved our knowledge about the neurobiological underpinnings of BPD [9], but the reports focusing on brain functional dynamics of this clinical picture in an early developmental stage are still very limited. Among brain imaging techniques, the electroencephalogram (EEG) is considered a suitable tool to investigate functional integration across brain areas providing valuable information about network dynamics related to BPD symptomatology [35]. More specifically, EEG connectivity is a neurophysiological index able to detect the specific electrophysiological signature that characterizes large-scale networks across different frequency bands [36,37]. Expanding the neurophysiological research on BPD to adolescent samples, especially by investigating neural dynamics during both RS and emotional/cognitive tasks, may be pivotal in reaching a better comprehension of the psychopathological processes involved in the early exhibition of this disorder as well as to add a developmental perspective to the literature on BPD. Furthermore, the investigation of EEG signatures that characterizes neural networks [36,37] is considered one of the most informative methods for the identification of brain abnormalities that can differentiate neuro-psychiatric from healthy populations [38]. Filling this gap may also provide useful bases for the development of preventive programs and tailored treatments addressed to adolescents.

Taking this into account, the aim of the current study was to extend previous literature analyzing EEG functional connectivity within SN hubs during RS and during the listening of two recordings of emotional autobiographical memories (i.e., happiness- and fear-related) in a sample of adolescents with BPD. In line with EEG findings in adult individuals with BPD [35], we hypothesized that adolescent patients would be characterized by disrupted neural connectivity during RS and by increased SN synchronization during the autobiographical negative emotional memory task.

## 2. Materials and Methods

### 2.1. Participants and Procedure

This study was conducted on 24 consecutive inpatients (mean age: 16.04 ± 1.26 years; 24 females) from the Inpatient Unit of Child and Adolescent Neuropsychiatry Section of “Sapienza” University of Rome (Department of Neuroscience and Mental Health) who received a BPD diagnosis, and 15 healthy controls (HCs; mean age: 16.57 ± 1.15 years; 14 females) recruited in the general population through community resources. This sample size was chosen according to previous EEG connectivity studies with adult BPD patients [35].

Participants were enrolled between September 2021 and September 2022. All participants underwent a sociodemographic and clinical assessment a week prior to the EEG recording session. The inclusion criteria for BPD patients were (i) a BPD diagnosis confirmed by the Structured Clinical Interview for DSM-5 Personality Disorders (SCID-5-PD; [39]); (ii) clinically relevant emotional dysregulation as confirmed by a Difficulties in Emotional Regulation Scale (DERS; [40]) score ≥127 [41]; (iii) negative anamnesis for a psychotic or bipolar disorder as confirmed by the Kiddie Schedule for Affective Disorders and Schizophrenia Present and Lifetime versions for DSM 5 (K-SADS-PL-5; [42]); (iv) age between 12 and 18 years; (v) right-handedness; (vi) negative anamnesis for neurological and cognitive disorders; (vii) parent’s and participant’s written informed consent to join the study. HCs inclusion criteria were (i) absence of any psychiatric disorder as confirmed by the SCID-5-PD and the K-SADS for DSM-5; (ii) absence of clinically relevant emotional dysregulation as confirmed by a DERS score <127 [41]; (iii) age between 12 and 18 years; (iv) right-handedness; (v) negative anamnesis for neurological and cognitive disorders; (vi) parent’s and participant’s written informed consent to join the study.

After the clinical assessment, participants were asked to recall and record a significant event of their life in which they experienced fear. Indeed, findings from previous studies suggest that people with BPD, or with heightened BPD features, may show particularly increased reactivity to fear-related stimuli (e.g., [43]). As a control emotional memory, they were also asked to recall and record a significant event of their life in which they experienced happiness. Memory recordings were collected using the built-in voice recorder of the smartphone.

The elapsed time until recall was measured and participants were also asked to report how old they were in each reported memory, and to rate on a 11-point Likert scale (0–10) the vividness of the recalled memory along with the intensity of the pleasure/distress experienced. In the following week, participants underwent an EEG recording, performed (i) in an RS-eyes closed condition, (ii) during the listening of their happiness-related memory recording (REC_H), and (iii) during the listening of their fear-related memory recording (REC_F). All participants listened to their own recordings via wired headphones. The order of the conditions was kept constant in order to avoid potential carry-over effects of the negative emotion elicited by the memory on the subsequent positive condition [44]. Furthermore, between the two listening conditions, they were given a 3 min rest. After each listening condition, participants were asked to rate again the vividness of the recalled memory and the intensity of the pleasure/distress experienced on an 11-point Likert scale. These subjective ratings were employed to assess the phenomenological quality and emotional impact of the recalled memories. The use of single-item scales has been previously employed in studies investigating autobiographical memory and emotional imagery in adolescent and adult populations [45]. While these scales are not standardized psychometric instruments, they may provide reliable indices of subjective experience and have demonstrated good sensitivity to emotional variations [46]. At the end of the experiment, participants followed an expert clinician for debriefing. All EEG recordings were performed under the same conditions for all participants. Researchers administering the EEG were not blinded to the participants’ assessment results.

A schematic representation of the experimental procedure is reported in Figure S1. Approval for the entire procedure was obtained from the Ethics Committee of the “Sapienza” University of Rome (Protocol No. 0001706) in accordance with the Helsinki Declaration guidelines.

### 2.2. Sociodemographic and Clinical Assessment

Participants underwent a clinical interview using the SCID-5-PD [39] and the K-SADS-PL-5 [42] performed by a trained psychologist. Current use of psychoactive substances (i.e., alcohol, nicotine, other illegal substances, psychiatric medications) was further noted apart, as well as the presence/absence of self-harm behaviors and suicidal attempts. The following questionnaires were also administered: the DERS [40], the Barratt Impulsiveness Scale Version 11 (BIS-11; [47]), the Dissociative Experiences Scale for Adolescents (A-DES; [48]), and the Childhood Trauma Questionnaire Short Form (CTQ-SF; [49]). This clinical assessment was conducted according to previous studies emphasizing the value of the combination of a categorical and psychopathological approach for the identification of BPD in adolescents [50]. A brief summary of questionnaires characteristics can be found in Table 1. Detailed description of the clinical assessment tools is reported in Supplementary Materials.

### 2.3. EEG Recordings

Participants were asked to abstain from caffeine, tobacco, or alcohol use at least 4 h before their EEG session. During all the EEG recordings, participants were sat on a comfortable chair with their eyes closed in the presence of a clinician. The first EEG recording consisted of a 5 min RS condition. During the second (REC_H) and third (REC_F) EEG recordings, participants listened to their happy and fear memory recording, respectively, with the use of headphones. Data were acquired using the Micromed System Plus digital EEGraph (Micromed© S.p.A., Mogliano Veneto, Italy), with a 31-electrode cap placed according to the International 10–20 System. A reference electrode was positioned on the right mastoid. Impedances were kept below 5 kΩ and were checked before and at the end of each EEG recording. The sampling rate was set at 256 Hz and the A/D conversion at 16 bits.

Using the EEGLAB toolbox for MatLab v.2022.1 [51], data were offline-filtered, setting the band-pass filters at 1–40 Hz, and the average reference was computed. A first visual inspection consented the removal of the most marked artifacts. Then, in order to remove other electrical, muscular, and visual artifacts, an independent component analysis (ICA) based on the infomax decomposition algorithm (“runica” tool of EEGLAB) was applied to all EEG channels (full details are described in [52]). Bad components were removed using the plug-in IClabel [53]. The EEG trace was further inspected for noisy channels from the data spectrum of the channels available in the “Plot” functions and a kurtosis of about five z-scores or more measured with the aid of the “Automatic channel rejection” function of EEGLAB. The main artifact-contaminated channels were subjected to a three-dimensional spherical spline interpolation [54,55].

### 2.4. EEG Functional Connectivity Analyses

**Figure 1:**
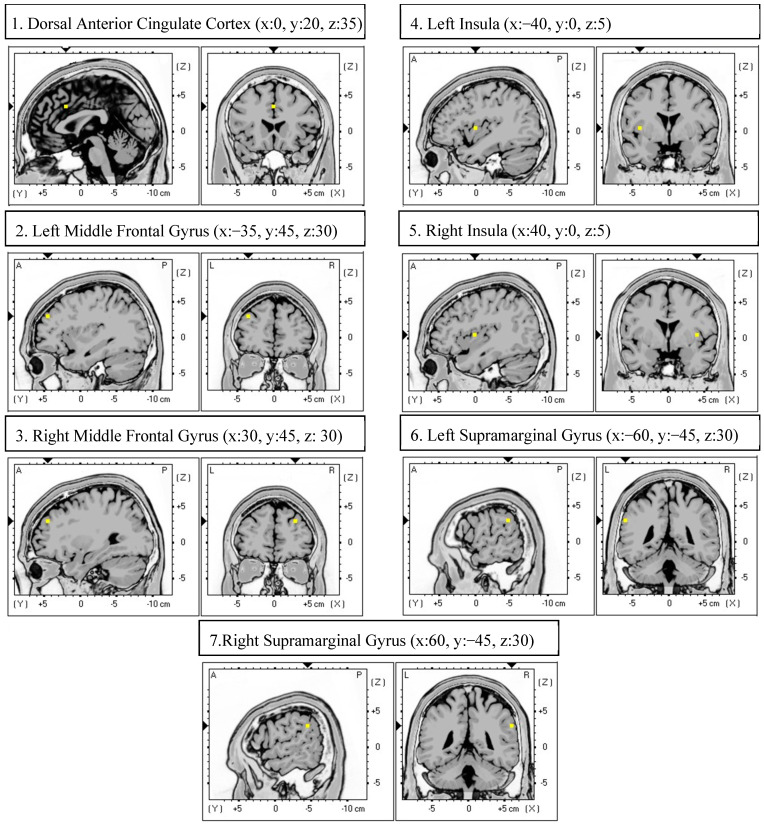
eLORETA Regions of Interest (i.e., yellow dot) of the salience network in sagittal and coronal views with Montreal Neurological Institute coordinates (x, y, z).

In accordance with previous EEG studies on adolescent samples [56,57], at least 60 s of the artifact-free EEG trace (not necessarily consecutive) were analyzed for each participant and for each condition (in the current study, the minimum length was 92 s). All EEG analyses were performed using the exact Low-Resolution Brain Electromagnetic Tomography software_v20190617_ (eLORETA; [58]), a validated software for functional connectivity analyses of large-scale brain networks [59]. eLORETA has been extensively used for its ability to accurately localize electrocortical activity even when recordings are performed with low-density electrode montages (i.e., <30) [60]. In order to run the EEG connectivity analysis, artifact-free data were fragmented into epochs of 4 seconds [61]. According to previous eLORETA studies [62,63,64], SN connectivity was investigated by defining seven Regions of Interest (ROIs; Figure 1) using the “ROI-maker#1” option available in the software. This approach uses the Montreal Neurological Institute (MNI) coordinates for the seed points employed to construct the target brain network. The connectivity analyses were performed by computing the Lagged Phase Synchronization (LPS; [58]), a measure of the similarity between signals of separate brain regions in each frequency domain based on a normalized Fourier transform. LPS values range between 0 (i.e., no synchronization) and 1 (i.e., maximum synchronization) and are computed after the removal of the instantaneous zero-lag contribution, which is liable to artifacts (e.g., volume conduction) and can lead to the detection of spurious functional coupling [65]. As in previous studies (e.g., [66,67]), the source reconstruction of the SN was made by selecting the eLORETA option “single nearest voxel”, so that each ROI consisted of the single voxel that was closest to each seed. Analyses were performed in the following frequency bands [68]: delta (1–4 Hz), theta (4.5–7.5 Hz), alpha (8–13 Hz), and beta (13.5–30 Hz).

### 2.5. Statistical Analyses

Statistical analyses were performed using the Statistical Package for the Social Sciences 25 (IBM, Armonk, NY, USA). Descriptive statistics were performed separately for BPD patients and HCs. Since several variables (e.g., DERS, CTQ, A-DES total scores) were not normally distributed (i.e., Shapiro–Wilk *p* < 0.05), group differences were examined using chi-square (χ^2^) tests for categorical variables and Kolmogorov–Smirnov tests for continuous variables. Functional connectivity analyses in the SN were performed both between groups (i.e., comparing BPD patients vs. HCs in each condition) and within groups (i.e., comparing RS vs. REC_H, RS vs. REC_F, and REC_H vs. REC_F in each group) for all ROIs in each frequency band. Specifically, we used the statistic non-parametric mapping available in the eLORETA software. This procedure consists of a modified version of Fisher’s test and applies non-parametric randomization to perform significance correction for multiple comparisons [69]. The “5000 randomizations” option was used to define the critical probability threshold of T-values corresponding to statistical *p*-values (*p* < 0.05 and *p* < 0.01) corrected for multiple testing [70]. The eLORETA software also calculates effect size (ES) thresholds for t-statistics corresponding to Cohen’s *d* values [71]: small = 0.2, medium = 0.5, large = 0.8. Finally, the association between clinical variables and any significant EEG connectivity results detected in the between/within-group comparisons was performed using Spearman’s rho correlations with 5000 bootstrap resamples and bias-corrected and accelerated (BCa) confidence intervals.

## 3. Results

In the current sample, no difference in age and sex was detected between BPD patients and HCs. Compared with HCs, BPD patients reported higher scores in all BPD-related dimensions, including self-report childhood trauma exposure (54.17 ± 15.54 vs. 29.87 ± 4.69; KS = 2.658; *p* < 0.001), dissociative symptoms (4.97 ± 2.38 vs. 2.42 ± 0.74; KS = 2.279; *p* < 0.001), impulsiveness (82.52 ± 11.00 vs. 63.47 ± 9.15; KS = 1.025; *p* = 0.001), and emotional dysregulation (148.75 ± 12.34 vs. 94.13 ± 19.61; KS = 3.038; *p* < 0.001).

Furthermore, BPD patients presented higher rates of alcohol (29.17% vs. 0%; χ^2^ = 5.332; *p* < 0.05), nicotine (66.67% vs. 13.33%; χ^2^ = 10.565; *p* < 0.01), and cannabis use (41.67% vs. 0%; χ^2^ = 8.405; *p* = 0.004) but not of use of other psychoactive substances. Complete descriptive statistics for sociodemographic and clinical variables are reported in Table 2.

For what concerns the characteristics of memories recalled, no between-group differences were detected in the memory-related age and elapsed time until recall, nor in the vividness and pleasure (for happiness) or distress (for fear) associated with the recall (neither in recording nor in listening). Data about recalled memories are reported in Table S1.

### 3.1. Between-Group Functional Connectivity Results

A qualitative visual inspection of the EEG recordings found no evidence of unusual patterns (e.g., sleepiness or epileptic discharges) in all participants. The effect sizes for the T-threshold were 1.217, 3.041, and 4.866, corresponding, respectively, to small (Cohen’s d value = 0.2), medium (Cohen’s d value = 0.5), and large (Cohen’s d value = 0.8) effect sizes.

**Figure 2:**
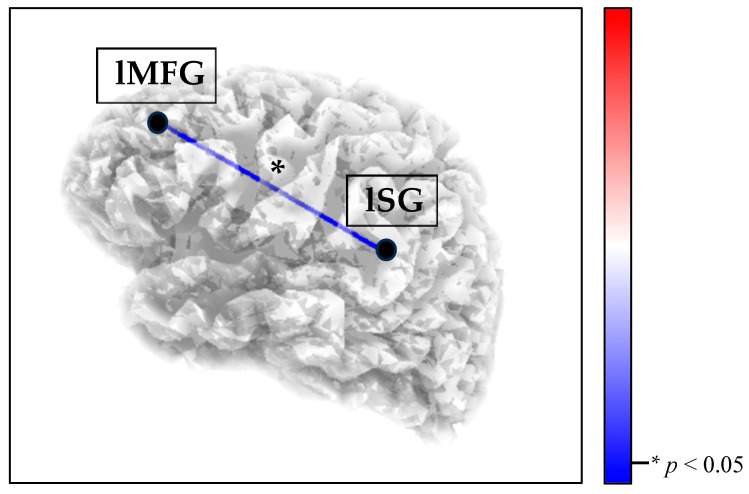
Decreased SN theta connectivity (blue line) between the left middle frontal gyrus (lMFG) and the left supramarginal gyrus (lSG) in the BPD group, compared with the HCs, during the RS condition. Red lines (not present) would indicate increased connectivity.

In the RS condition, the eLORETA thresholds for significance (corrected for multiple comparisons) were T = ±4.281 (corresponding to *p* = 0.01) and T = ±3.662 (corresponding to *p* = 0.05). Significant differences between groups were observed in the theta band. Compared to the HCs, the BPD group showed a decrease in theta connectivity between the left middle frontal gyrus and the left supramarginal gyrus (T = −3.672, *p* = 0.049; Figure 2).

No significant between-group differences were detected in the other frequency bands nor comparing the other conditions. Complete matrices can be found in Figures S2–S4.

#### Within Functional Connectivity Results

For the BPD group, a significant difference in the SN functional connectivity was detected only in the RS vs. REC_F comparison. The effect sizes for the T-threshold were 0.938, 2.345, and 3.752, corresponding, respectively, to small (Cohen’s d value = 0.2), medium (Cohen’s d value = 0.5), and large (Cohen’s d value = 0.8) effect sizes. In this comparison, the eLORETA thresholds for significance (corrected for multiple comparisons) were T = ±4.457 (corresponding to *p* = 0.01) and T = ±3.817 (corresponding to *p* = 0.05).

**Figure 3:**
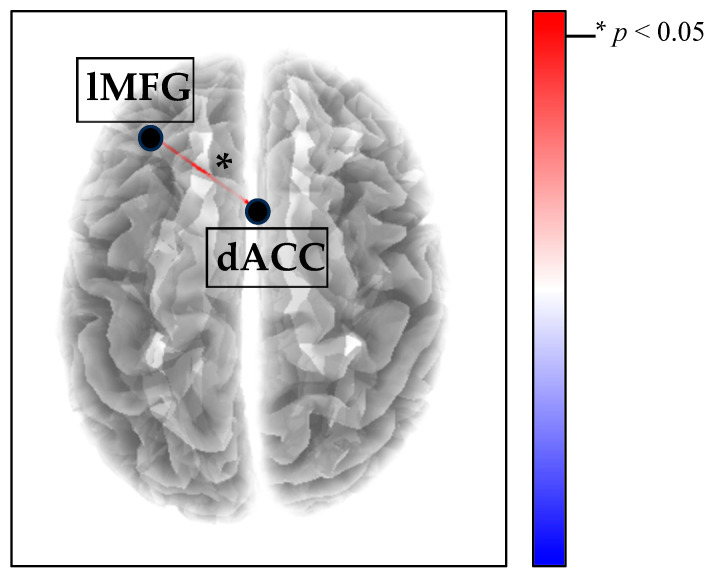
Increased SN delta connectivity (red line) between the left middle frontal gyrus (lMFG) and the dorsal anterior cingulate cortex (dACC) in the REC_F condition compared with the RS in the BPD group. Blue lines (not present) would indicate decreased connectivity.

Compared with the RS condition, in the REC_F condition, BPD patients showed an increase in delta connectivity between the left middle frontal gyrus and the anterior cingulate cortex (T = 3.924; *p* = 0.019; Figure 3). No significant within-group differences were detected in the other frequency bands nor comparing the other conditions.

In the HC group, no significant difference in the SN connectivity among all conditions was detected. Complete matrices can be found in Figures S5–S10.

### 3.2. Correlations

RS theta functional connectivity between the left middle frontal gyrus and left supramarginal gyrus was negatively correlated with all BPD-related dimensions (Table 3).

For what concerns the within-group analysis, delta functional connectivity of the left middle frontal gyrus and anterior cingulate cortex between REC_F and RS was negatively correlated with the vividness of the recalled memory (*rho* = −0.450; *p* < 0.05; N = 24), indicating that the greater the delta SN connectivity in REC_F compared with RS, the less the vividness of the memory. No other significant correlations between functional connectivity and clinical variables were observed.

## 4. Discussion

The aim of this study was to investigate the SN functional connectivity during the resting state (RS) and during the listening of the recording of two emotional autobiographical memories in a sample of BPD adolescents. Compared with HCs, BPD patients reported reduced theta SN connectivity (i.e., between the left middle frontal gyrus and the left supramarginal gyrus) during RS. Further analyses showed that RS theta functional connectivity in these brain areas was negatively correlated with all BPD-related dimensions (i.e., emotional dysregulation, impulsiveness, dissociative symptoms, and childhood trauma) in the whole sample. Finally, compared with RS, BPD patients, but not HCs, showed increased delta SN connectivity (i.e., between the left middle frontal gyrus and the dACC) during REC_F. The difference in delta SN connectivity between the two conditions (i.e., REC_F and RS) was negatively correlated with the self-report vividness of the recall.

The SN is involved in the attentional control function, detecting salient stimuli, switching between other large-scale brain networks, and leading to appropriate responses [72]. Disruption of the SN has been associated with several psychiatric disorders [26], including BPD [29,30,31,33]. However, this is one of the first studies to directly explore EEG SN connectivity in an adolescent BPD sample.

Coherently with previous findings on adult BPD samples in RS [30,31,35], the current study first found reduced SN connectivity in BPD adolescents, compared with HCs. Decreased functional connectivity was observed between the left middle frontal gyrus and the left supramarginal gyrus. The left middle frontal gyrus has been widely associated with the attentional reorientation function [73], while the left supramarginal gyrus is thought to be involved in the processing of external/perceptual information, mediating the external-oriented attention to contingent world [74]. It is worth noting that the functional connectivity reduction between these brain areas was detected in the theta band, which is considered to play a primary role in long-distance integration of brain regions [75], sustaining attentional cognitive processes [76]. Therefore, reduced theta connectivity between the left middle frontal gyrus and the left supramarginal gyrus may reflect an impairment in the integration of salient perceptual information to redirect attention towards the external world with appropriate self-regulation strategies. Coherently, the theta functional connectivity between the left middle frontal gyrus and the left supramarginal gyrus was negatively correlated with self-report childhood trauma, which have been suggested to lead to the disintegrative pathogenic processes that underlie several dissociative manifestations and can affect emotional regulation abilities and impulse control [34,77,78,79]. These latter were negatively correlated with functional connectivity as well, suggesting that the hypo-connection between the middle frontal gyrus and the supramarginal gyrus may be a common neural marker of the main BPD-related dimensions.

The opposite connectivity pattern (i.e., increased SN functional connectivity) in BPD patients was detected during the listening of their own recording of fear-related autobiographical memories compared with the RS condition. This findings may be consistent with a previous study observing hyperactivity of these SN nodes in BPD samples during exposure to emotional autobiographical memories [80,81]. The dACC is considered to be involved in the processing of negative emotions [82] and to play a significant role in demanding for cognitive control over negative emotions from frontal areas [83]. In particular, recent studies suggest that the connection of the dACC with frontal brain regions can be implicated in the repression of negative emotions [84] and the suppression of unwelcome memories [85]. Furthermore, the functional hyperconnectivity between the anterior cingulate cortex and left middle frontal gyrus was detected in the delta band, which has been shown to support proactive cognitive control [86,87] and autobiographical memory [88] processes. Therefore, it may be speculated that increased delta functional connectivity between these brain areas may reflect the higher effort to suppress unwanted and distressing memories. Of relevance, we detected a negative correlation between this hyper-connectivity pattern during the task and the vividness of the memory, suggesting a possible effort to control its negative impact. It is worth noting that there were no group differences in the subjective distress or vividness associated with the fear-related memory, suggesting that increased delta connectivity in the BPD group is not linked to higher perceived distress or intensity of the recall, but may effectively depend on the unbearableness of negative memories for BPD patients leading to a possible suppression mechanism. Consistently, previous studies found that adolescents with BPD traits exhibit a higher use of suppression and poorer use of reappraisal strategies in coping with negative emotions [89,90].

However, these interpretations are merely speculative, and further studies are needed in order to confirm and extend the comprehension of the results. The current study, moreover, has some limitations to be considered. First, as a result of consecutive recruitment in our clinical unit, all BPD participants were female. This overrepresentation of female patients is consistent with clinical and epidemiological data showing that women are more frequently diagnosed with BPD in treatment-seeking populations [91,92]. Previous research has also shown that BPD often presents differently in men and women: for example, females tend to exhibit more internalizing symptoms such as affective instability and self-harm, whereas males more frequently display externalizing behaviors like aggression or substance misuse [91,92]. Considering these differences in clinical presentation, it is plausible that underlying physiological mechanisms may also vary by sex. Consequently, our results should be interpreted with caution, as the physiological patterns identified here may not fully generalize to male individuals with BPD. A larger and more balanced sample is advisable in future studies. As a second limitation, the fixed task order (happiness followed by fear) may be subject to an order-effect bias. Moreover, we used a 31-channel EEG, which has an intrinsic limit in space resolution and localization accuracy compared to high-density tools [93]. Another important limitation of the current study is related to the high rates of patients with psychiatric comorbidities and psychotropic medication use. Although this is a common issue in EEG studies with BPD patients, it makes it challenging to determine whether the observed electrophysiological patterns are attributable to the core characteristics of BPD or are instead shaped by comorbid disorders and/or medications [35]. Finally, the current study was limited to the comparison of HCs and BPD adolescents, as well as to the comparison of RS with happiness and fear autobiographical memory recalls. Future investigations may include a sample of psychiatric adolescents patients without BPD, in order to discriminate whether the results are specific to BPD or to cross-cutting symptom dimensions and may further analyze functional connectivity during the recall of different negative emotions, such as sadness or anger. Therefore, overall, the results of the current report should be considered preliminary and treated with caution, and future longitudinal studies are needed to establish whether these neural patterns can serve as reliable biomarkers or therapeutic targets.

Despite this, to our knowledge, this is the first study to extensively investigate the SN functional connectivity in a sample of BPD adolescents in RS and during two types of emotional autobiographical memory recall. Findings show that, during RS, BPD patients exhibit reduced theta SN connectivity, compared with HCs. On the contrary, they report increased delta SN connectivity during REC_F, compared with RS, and this hyper-connectivity pattern is negatively correlated with the self-report vividness of the recall. While the decreased SN theta connectivity may be a common neural marker of disintegration associated with the main BPD-related dimensions (i.e., emotional dysregulation, impulsiveness, dissociation and childhood trauma), the increased SN delta connectivity may reflect a suppression mechanism for unbearable fear-related memories.

## 5. Conclusions

To summarize, our study showed that female adolescents with BPD are characterized by (i) disrupted SN neural connectivity during RS (Figure 2) and (ii) increased SN synchronization during the listening of their own fear-related autobiographical memory task (Figure 3). This study extends the limited literature on adolescent neural correlates of BPD, potentially leading to a better understanding of the neurophysiological mechanisms underlying the early manifestation of the disorder. By incorporating a developmental perspective into our understanding of BPD neural markers, this research could have potential clinical implications. Specifically, given the high-risk profile of BPD adolescents, it is crucial to develop and enhance the effectiveness of therapy programs. Our findings seem to support the disintegrative nature of BPD pathological dimensions, highlighting the importance of addressing these processes and advocating the clinical usefulness of functional connectivity data to monitor treatment quality in adolescents with BPD. EEG functional connectivity, in particular, is emerging as a valuable tool for assessing therapy outcomes, especially in developmental trauma-based pathologies such as BPD [94,95,96]. Additionally, this study sheds light on the neurophysiological processes involved in autobiographical memory recall, a key component of psychotherapy for BPD adolescents, suggesting that the recall of fear-related memories may be processed differently in BPD adolescents and might require specific therapeutic strategies.

## Figures and Tables

**Table 1:** Summary of the main characteristics of the assessment instruments.

Instrument	Type	Brief Description
SCID-5-PD	Clinical Interview	Semi-structured clinical interview used to assess personality disorders according to DSM-5 criteria.
K-SADS-PL-5	Clinical Interview	Semi-structured interview for children and adolescents assessing current and past psychiatric disorders according to DSM-5 criteria.
DERS	Self-report	36-item questionnaire assessing difficulties in emotion regulation across multiple domains.
BIS-11	Self-report	30-item questionnaire measuring impulsivity in adults across attentional, motor, and non-planning domains.
A-DES	Self-report	30-item questionnaire assessing dissociative experiences in adolescents.
CTQ-SF	Self-report	28-item questionnaire measuring childhood abuse and neglect, including a scale to detect underreporting.

Abbreviations: SCID-5-PD = Structured Clinical Interview for DSM-5 Personality Disorders; K-SADS-PL-5 = Kiddie Schedule for Affective Disorders and Schizophrenia Present and Lifetime versions for DSM-5; DERS = Difficulties in Emotion Regulation Scale; BIS-11 = Barratt Impulsiveness Scale-11; A-DES = Dissociative Experiences Scale for Adolescents; CTQ-SF = Childhood Trauma Questionnaire-Short Form.

**Table 2:** Descriptive statistics of sociodemographic and clinical variables with group difference tests.

Variables	BPD (N = 24)	HCs (N = 15)	Test	*p*
Age—M ± SD	16.04 ± 1.26	16.57 ± 1.15	KS = 1.063	*p* = 0.208
Females—N (%)	24 (100)	14 (93.33)	χ^2^ = 1.642	*p* = 0.200
Alcohol use—N (%)	7 (29.17)	0 (0)	χ^2^ = 5.332	***p* = 0.021**
Nicotine use—N (%)	16 (66.67)	2 (13.33)	χ^2^ = 10.565	***p* = 0.001**
Cannabis use—N (%)	10 (41.67)	0 (0)	χ^2^ = 8.405	***p* = 0.004**
Other substances use—N (%)	1 (4.17)	0 (0)	χ^2^ = 0.641	*p* = 0.423
Self-harm—N (%)	24 (100)	-	-	-
Suicidal risk				
Suicidal ideation N (%)	13 (54.17)	-	-	-
Suicidal attempt N (%)	10 (41.67)	-	-	-
Other psychiatric disorders * N (%)	23 (95.83)	-	-	-
Psychiatric medication use—N (%)	22 (91.67)	-	-	-
DERS—M ± SD	148.75 ± 12.34	94.13 ± 19.61	KS = 3.038	***p <* 0.001**
BIS-11—M ± SD	82.52 ± 11.00	63.47 ± 9.15	KS = 1.025	***p =* 0.001**
Attentional impulsivity—M ± SD	23.09 ± 4.27	17.53 ± 2.72	KS = 1.177	***p <* 0.001**
Motor—M ± SD	27.26 ± 4.61	19.80 ± 3.39	KS = 1.899	***p =* 0.001**
Non-planning—M ± SD	32.13 ± 4.36	26.20 ± 4.92	KS = 1.696	***p* = 0.006**
A-DES—M ± SD	4.97 ± 2.38	2.42 ± 0.74	KS = 2.279	***p <* 0.001**
CTQ-SF—M ± SD	54.17 ± 15.54	29.87 ± 4.69	KS = 2.658	***p <* 0.001**
PA—M ± SD	6.52 ± 2.28	5.20 ± 0.56	KS = 0.987	*p* = 0.284
EA—M ± SD	14.87 ± 5.29	6.27 ± 1.94	KS = 2.532	***p <* 0.001**
SA—M ± SD	9.70 ± 6.03	5.00 ± 0.00	KS = 1.646	***p* = 0.009**
PN—M ± SD	7.44 ± 2.62	5.40 ± 0.63	KS = 1.266	***p* = 0.081**
EN—M ± SD	15.65 ± 4.99	8.00 ± 2.51	KS = 2.253	***p <* 0.001**
MD—M ± SD	0.46 ± 1.06	0.47 ± 0.92	KS = 0.304	*p* = 1.000

Abbreviations: A-DES = Dissociative Experiences Scale for Adolescents; BIS-11 = Barratt Impulsiveness Scale-11; CTQ-SF = Childhood Trauma Questionnaire-Short Form; DERS = Difficulties in Emotion Regulation Scale; EA = emotional abuse; EN = emotional neglect; KS = Kolmogorov–Smirnov test; MD = minimization/denial; PA = physical abuse; PN = physical neglect; SA = sexual abuse. * Current or past diagnoses, which included attention deficits and hyperactivity disorder, oppositional defiant disorder, depressive disorders, anxiety disorders, substance use disorders, eating disorders, and post-traumatic stress disorder. Notes: *p* values under 0.05 are **bolded**.

**Table 3:** Spearman’s *rho* correlations among significant data of functional connectivity resulted from the *between-group* analysis and clinical variables (whole sample).

		1.	2.	3.	4.
1. A-DES	*rho*	-			
	*p*	-			
2. CTQ-SF	*rho*	0.450	-		
	*p*	**0** **.005**	-		
3. BIS-11	*rho*	0.303	0.671	-	
	*p*	0.068	**<** **0** **.001**	-	
4. DERS	*rho*	0.570	0.743	0.649	-
	*p*	**<** **0** **.001**	**<** **0** **.001**	**<** **0** **.001**	-
5. RS theta FC between lMFG and lSG	*rho*	−0.414	−0.479	−0.431	−0.520
	*p*	**0** **.010**	**0** **.002**	**0** **.007**	**0** **.001**

Abbreviations: A-DES = Dissociative Experiences Scale for Adolescents; BIS-11 = Barratt Impulsiveness Scale-11; CTQ-SF = Childhood Trauma Questionnaire-Short Form; DERS = Difficulties in Emotion Regulation Scale; FC = functional connectivity; lMFG = left middle frontal gyrus; lSG = left supramarginal gyrus; RS = resting-state. Notes: *p* values under 0.05 are **bolded**.

## Data Availability

The raw data supporting the conclusions of this article will be made available by the authors on request due to restrictions (privacy reasons).

## Supplementary Materials

The following supporting information can be downloaded at https://www.mdpi.com/article/10.3390/brainsci15111146/s1. Figure S1. Flowchart showing the experimental procedure. Assessment instruments description. Table S1. Descriptive statistics for memory characteristics with group difference tests. Figure S2. The complete T-value matrix of functional connectivity results of between-group comparisons (BPD vs. HCs) for the RS condition in all frequency bands (N = 39). Figure S3. The complete T-value matrix of functional connectivity results of between-group comparisons (BPD vs. HCs) for the REC_H condition in all frequency bands (N = 39). Figure S4. The complete T-value matrix of functional connectivity results of between-group comparisons (BPD vs. HCs) for the REC_F condition in all frequency bands (N = 39). Figure S5. The complete T-value matrix of functional connectivity results of within-group (BPD) comparison (REC_H vs. RS) in all frequency bands (N = 24). Figure S6. The complete T-value matrix of functional connectivity results of within-group (BPD) comparison (REC_F vs. RS) in all frequency bands (N = 24). Figure S7. The complete T-value matrix of functional connectivity results of within-group (BPD) comparison (REC_F vs. REC_H) in all frequency bands (N = 24). Figure S8. The complete T-value matrix of functional connectivity results of within-group (HC) comparison (REC_H vs. RS) in all frequency bands (N = 15). Figure S9. The complete T-value matrix of functional connectivity results of within-group (HC) comparison (REC_F vs. RS) in all frequency bands (N = 15). Figure S10. The complete T-value matrix of functional connectivity results of within-group (HC) comparison (REC_F vs. REC_H) in all frequency bands (N = 15).
