# Supplementary material for: Aberrant Salience Network Functional Connectivity in Resting-State and Fear-Related Autobiographical Memory Recall in Female Adolescents with Borderline Personality Disorder
Source: Brain Sci. 2025 Oct 25;15(11):1146. doi: 10.3390/brainsci15111146 (PMC12650605; doi:10.3390/brainsci15111146)
Supplement: Supplementary file 1 [file brainsci-15-01146-s001.zip › brainsci-3929069-supplementary.pdf]

Figure S1. Flowchart showing the experimental procedure.

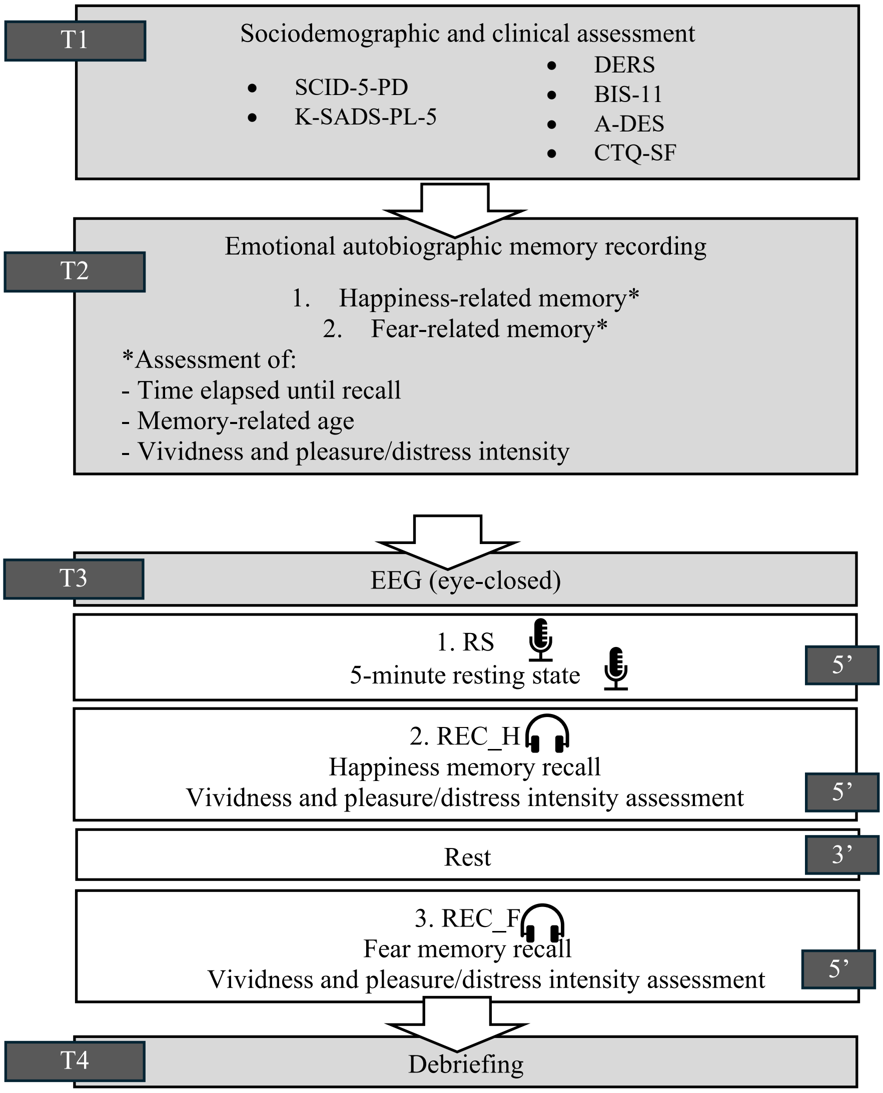

Abbreviations: SCID-5-PD=Structured Clinical Interview for DSM-5 Personality Disorders; K-SADS-PL-5=Kiddie Schedule for Affective Disorders and Schizophrenia Present and Lifetime versions for DSM-5; DERS=Difficulties in Emotion Regulation Scale; BIS-11=Barratt Impulsiveness Scale-11; A-DES=Dissociative Experiences Scale for Adolescents; CTQ-SF=Childhood Trauma Questionnaire-Short Form.

Assessment instruments description

SCID-5-PD

The SCID-5-PD [1] is a semi-structured diagnostic interview developed to assess the Personality Disorders as classified in the categorical model of the DSM-5. The SCID-5-PD consists of 90 questions scored on a three-point scale indicating “absent,” “subthreshold,” and “present”. Despite questions can be asked consecutively, administrators are encouraged to follow patients’ responses and ask for concrete examples that can support them. The decision on the fulfillment of the scoring criteria is up to the clinician, so that clinical knowledge and experience is more relevant than in other assessment measures (e.g. self-report and fully structured interviews). The interview has been previously used in adolescent samples [e.g.

2]. In the current study, an expert and trained clinician was responsible for the administration of the Italian version of the interview [3].

K-SADS-PL-5

The K-SADS-PL-5 [4] is a semi-structured diagnostic interview designed to assess general psychopathology in child or adolescents in accordance with DSM-5 criteria. The interviewer can collect information from the child/adolescent him/herself as well as from parents or other informants. The K-SADS-PL-5 consists of a screening section that covers the primary symptoms of each disorder and six supplements. Whether at least one symptom of a disorder receives positive answer, the assessment of the disorder is further investigated in the corresponding supplement. Supplements include: (i) depressive and bipolar disorders; (ii) psychotic disorders; (iii) anxiety, stress and obsessive compulsive disorders; (iv) disruptive behavior and impulse control disorders; (v) substance use disorders and feeding and eating disorders; (vi) neurodevelopmental disorders. The interview consent to assess both current episodes (i.e. within the last six months) and past episodes. In the current study the Italian translation of the interview was used by an expert and trained clinician [5].

DERS

The DERS [6] is a validated 36-items questionnaire that assesses the impairment in six emotional regulation domains: (i) nonacceptance of emotional response; (ii) difficulties in adopting goal-directed behaviors; (iii) difficulties in controlling impulsive behaviors; (iv) lack of emotional awareness; (v) limited access to emotion regulation strategies, and (vi) lack of emotional identification or clarity. All items are scored on a five-point Likert scale from 1= “almost never” to 5= “almost always”, with higher scores indicating higher emotional dysregulation. The questionnaire has been validated in adolescents samples [7] and a previous study suggested that DERS score can distinguish BPD adolescent patients from non-BPD psychiatric patients and healthy controls [8]. Although an official cut-off is not available yet, we set a score of 127 to distinguish BPD adolescents from healthy controls on the base of a previous study [9]. We used the Italian version of the instrument [10].

BIS-11

The BIS-11 [11] is one of the most used tools to assess impulsivity, demonstrating high validity, reliability, and predictive value [12]. The BIS-11 consists of 30 items scored on a 5-points Likert scale ranging from 1=“never” to 4=“very frequently”. It assesses three main facets of impulsive behavior: attentional (i.e. a lack of focus on the ongoing task), motor (i.e. acting without thinking), and non-planning (i.e. lack of consideration of the future) impulsivity. Higher scores imply higher impulsivity. The questionnaire has been validated in adolescent samples [13]. The Italian version of the questionnaire has been used in the current study [14].

A-DES

The A-DES [15] is a 30-item validated questionnaire adapted to measure dissociative experiences (i.e. absorption, depersonalization and derealization, dissociative amnesia and passive influence) in adolescents. Each item is rated on a 11-point Likert scale ranging from 0= "never" to 10= "always", indicating the frequency of the dissociative experience described by each item. The total score is obtained by averaging across item scores. In the current study the Italian version of the instrument was used [16].

CTQ-SF

The CTQ-SF [17] is a widely validated 28-item scale assessing exposure to the five main types of traumatic childhood experiences, namely: physical abuse, emotional abuse, sexual abuse, physical neglect and emotional neglect. Each item is rated on a 5-point Likert scale ranging from 1="Never true" to 5= "Very often true". Therefore, the score per subscale ranges from a minimum of 5 to a maximum of 25, while the total score ranges between 25 and 125. Higher scores reflect greater severity of childhood traumatic experiences. The questionnaire implies an additional 3-item scale that measures Minimization/Denial (M/D), whose total score ranges from 0 to 3. While the psychometric properties of the questionnaire in adolescent samples are consistent, some caution is advised, since available data suggest that test-retest stability is weaker and the interpretation of the M/D scale is ambiguous [18]. In the current study the Italian version of the instrument was used [19].

Table S1. Descriptive statistics for memories characteristics with group differences tests.

**Variables**	**BPD (N=24)** M ± SD	**HCs (N=15)** M ± SD	**Test**	***p***
**Happiness-related memory**				
Time elapsed until recall	29.75 ± 24.30	19.29 ± 17.16	KS=1.013	*p*=0.257
Memory-related age	13.08 ± 3.02	13.47 ± 3.02	KS=0.582	*p*=0.887
Vividness (in recording)	7.88 ± 1.80	8.00 ± 1.41	KS=0.228	*p*=1.000
Vividness (in listening)	7.79 ±1.72	7.73 ± 1.28	KS=0.506	*p*=0.960
Pleasure (in recording)	8.33 ± 2.20	9.00 ± 1.00	KS=0.532	*p*=0.948
Pleasure (in listening)	8.13 ± 1.73	8.87 ± 0.99	KS=0.706	*p*=0.696
**Fear-related memory**				
Time elapsed until recall	19.46 ± 24.65	35.13 ± 41.17	KS=1.063	*p*=0.208
Memory-related age	13.21 ± 2.64	14.80 ± 1.70	KS=1.165	*p*=0.133
Vividness (in recording)	8.33 ± 1.79	8.07 ± 1.53	KS=0.582	*p*=0.887
Vividness (in listening)	8.42 ± 1.82	8.13 ± 1.46	KS=0.633	*p*=0.818
Distress (in recording)	9.08 ± 1.47	8.20 ± 1.52	KS=1.114	*p*=0.167
Distress (in listening)	8.71 ± 1.83	7.93 ± 1.62	KS=1.013	*p*=0.257

Abbreviations: KS= Kolmogorov-Smirnov test
Note: Time is reported in seconds

Figure S2. The complete T-values matrix of functional connectivity results of between-groups comparisons (BPD vs HCs) for the RS condition in all frequency bands (N=39).

BPD vs HC in RS

Delta (1-4 Hz)

	dACC	lMFG	rMFG	lIns	rIns	lSMG	rSMG
dACC	0.000	-2.331	-1.670	-0.752	-2.302	-2.008	-1.441
lMFG	-2.331	0.000	-1.614	-1.520	-1.830	-2.075	-2.206
rMFG	-1.670	-1.614	0.000	-0.668	-1.348	-2.668	-0.690
lIns	-0.752	-1.520	-0.668	0.000	-0.394	-2.322	0.980
rIns	-2.302	-1.830	-1.348	-0.394	0.000	-2.019	-0.833
lSMG	-2.008	-2.075	-2.668	-2.322	-2.019	0.000	-0.443
rSMG	-1.441	-2.206	-0.690	0.980	-0.833	-0.443	0.000

Theta (4.5-7.5 Hz)

	dACC	lMFG	rMFG	lIns	rIns	lSMG	rSMG
dACC	0.000	-0.260	-0.201	-0.634	-1.471	-1.284	0.213
lMFG	-0.260	0.000	-1.024	-0.305	-1.190	**-3.672**	-1.198
rMFG	-0.201	-1.024	0.000	-1.637	-1.186	-1.350	-1.163
lIns	-0.634	-0.305	-1.637	0.000	-0.318	-2.307	-0.994
rIns	-1.471	-1.190	-1.186	-0.318	0.000	-1.437	-1.295
lSMG	-1.284	**-3.672**	-1.350	-2.307	-1.437	0.000	-0.705
rSMG	0.213	-1.198	-1.163	-0.994	-1.295	-0.705	0.000

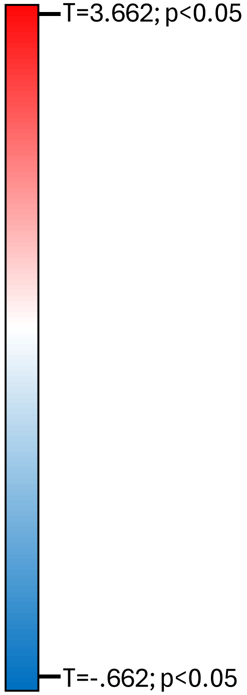

Alpha (8–13 Hz)

	dACC	lMFG	rMFG	lIns	rIns	lSMG	rSMG
dACC	0.000	0.783	-0.862	0.673	-0.863	0.064	1.158
lMFG	0.783	0.000	0.486	0.595	-0.261	-0.931	-1.260
rMFG	-0.862	0.486	0.000	0.313	-0.197	-0.362	-0.128
lIns	0.673	0.595	0.313	0.000	0.927	-1.063	-0.122
rIns	-0.863	-0.261	-0.197	0.927	0.000	-1.740	-0.810
lSMG	0.064	-0.931	-0.362	-1.063	-1.740	0.000	-0.853
rSMG	1.158	-1.260	-0.128	-0.122	-0.810	-0.853	0.000

Beta (13.5–30 Hz)

	dACC	lMFG	rMFG	lIns	rIns	lSMG	rSMG
dACC	0.000	2.125	1.545	1.240	0.310	1.466	1.436
lMFG	2.125	0.000	0.115	0.532	0.208	1.244	-0.481
rMFG	1.545	0.115	0.000	0.142	-0.044	-0.612	0.631
lIns	1.240	0.532	0.142	0.000	0.401	0.232	0.210
rIns	0.310	0.208	-0.044	0.401	0.000	0.157	0.337
lSMG	1.466	1.244	-0.612	0.232	0.157	0.000	-0.004
rSMG	1.436	-0.481	0.631	0.210	0.337	-0.004	0.000

Abbreviations: dACC: dorsal Anterior Cingulate Cortex; lMFG: left Middle Frontal Gyrus; rMFG: right Middle Frontal Gyrus; lIns: left Insula; rIns: right Insula; lSMG: left Supramarginal Gyrus; rSMG: right Supramarginal Gyrus

Figure S3. The complete T-values matrix of functional connectivity results of between-groups comparisons (BPD vs HCs) for the REC_H condition in all frequency bands (N=39).

BPD vs HC in REC_H

Delta (1-4 Hz)

	dACC	lMFG	rMFG	lIns	rIns	lSMG	rSMG
dACC	0,000	-1,142	-1,549	-0,596	-0,695	-1,396	-1,339
lMFG	-1,142	0,000	-1,629	0,221	-0,283	-1,074	-0,690
rMFG	-1,549	-1,629	0,000	-0,439	0,475	-0,510	-0,297
lIns	-0,596	0,221	-0,439	0,000	0,647	-1,242	0,194
rIns	-0,695	-0,283	0,475	0,647	0,000	-1,586	-0,638
lSMG	-1,396	-1,074	-0,510	-1,242	-1,586	0,000	-1,555
rSMG	-1,339	-0,690	-0,297	0,194	-0,638	-1,555	0,000

Theta (4.5-7.5 Hz)

	dACC	lMFG	rMFG	lIns	rIns	lSMG	rSMG
dACC	0,000	1,911	0,235	1,939	1,412	0,351	0,614
lMFG	1,911	0,000	-0,529	1,926	0,522	-0,414	-0,203
rMFG	0,235	-0,529	0,000	0,893	1,576	0,194	1,318
lIns	1,939	1,926	0,893	0,000	0,161	-0,217	0,393
rIns	1,412	0,522	1,576	0,161	0,000	0,897	0,274
lSMG	0,351	-0,414	0,194	-0,217	0,897	0,000	1,103
rSMG	0,614	-0,203	1,318	0,393	0,274	1,103	0,000

Alpha (8–13 Hz)

	dACC	lMFG	rMFG	lIns	rIns	lSMG	rSMG
dACC	0,000	1,763	1,977	1,465	0,908	0,417	1,236
lMFG	1,763	0,000	1,348	0,691	-0,828	-0,907	-0,025
rMFG	1,977	1,348	0,000	1,468	0,805	3,074	2,776
lIns	1,465	0,691	1,468	0,000	-0,693	-1,147	1,526
rIns	0,908	-0,828	0,805	-0,693	0,000	1,836	1,225
lSMG	0,417	-0,907	3,074	-1,147	1,836	0,000	-0,309
rSMG	1,236	-0,025	2,776	1,526	1,225	-0,309	0,000

Beta (13.5–30 Hz)

	dACC	lMFG	rMFG	lIns	rIns	lSMG	rSMG
dACC	0,000	1,759	0,925	1,605	1,163	1,505	0,718
lMFG	1,759	0,000	-0,799	-1,381	0,559	-1,181	-0,135
rMFG	0,925	-0,799	0,000	0,223	0,054	-1,042	-0,058
lIns	1,605	-1,381	0,223	0,000	0,162	-0,032	-0,104
rIns	1,163	0,559	0,054	0,162	0,000	0,060	1,446
lSMG	1,505	-1,181	-1,042	-0,032	0,060	0,000	-0,101
rSMG	0,718	-0,135	-0,058	-0,104	1,446	-0,101	0,000

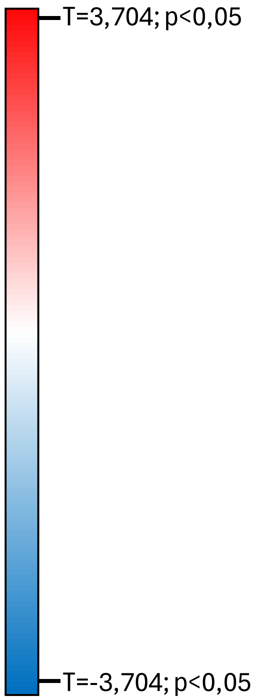

Abbreviations: dACC: dorsal Anterior Cingulate Cortex; lMFG: left Middle Frontal Gyrus; rMFG: right Middle Frontal Gyrus; lIns: left Insula; rIns: right Insula; lSMG: left Supramarginal Gyrus; rSMG: right Supramarginal Gyrus

Figure S4. The complete T-values matrix of functional connectivity results of between-groups comparisons (BPD vs HCs) for the REC_F condition in all frequency bands (N=39).

BPD vs HC in REC_F

Delta (1-4 Hz)

	dACC	lMFG	rMFG	lIns	rIns	lSMG	rSMG
dACC	0.000	0.549	-0.815	0.290	0.086	0.608	0.754
lMFG	0.549	0.000	-1.008	-0.726	-0.077	-2.250	-0.248
rMFG	-0.815	-1.008	0.000	-1.031	-0.075	-0.449	-0.131
lIns	0.290	-0.726	-1.031	0.000	-1.160	0.122	-0.368
rIns	0.086	-0.077	-0.075	-1.160	0.000	-0.496	0.900
lSMG	0.608	-2.250	-0.449	0.122	-0.496	0.000	-0.704
rSMG	0.754	-0.248	-0.131	-0.368	0.900	-0.704	0.000

Theta (4.5-7.5 Hz)

	dACC	lMFG	rMFG	lIns	rIns	lSMG	rSMG
dACC	0.000	0.897	1.374	1.903	0.890	0.542	1.162
lMFG	0.897	0.000	-0.167	0.043	0.333	0.525	1.619
rMFG	1.374	-0.167	0.000	1.193	-1.234	2.616	1.464
lIns	1.903	0.043	1.193	0.000	1.806	0.410	1.176
rIns	0.890	0.333	-1.234	1.806	0.000	1.802	0.260
lSMG	0.542	0.525	2.616	0.410	1.802	0.000	1.308
rSMG	1.162	1.619	1.464	1.176	0.260	1.308	0.000

Alpha (8–13 Hz)

	dACC	lMFG	rMFG	lIns	rIns	lSMG	rSMG
dACC	0.000	0.293	-0.382	0.189	-0.066	-0.940	1.232
lMFG	0.293	0.000	1.590	0.758	-0.493	-1.225	-0.016
rMFG	-0.382	1.590	0.000	0.293	-0.300	-0.763	1.046
lIns	0.189	0.758	0.293	0.000	-1.042	-0.285	0.780
rIns	-0.066	-0.493	-0.300	-1.042	0.000	0.520	-0.211
lSMG	-0.940	-1.225	-0.763	-0.285	0.520	0.000	0.400
rSMG	1.232	-0.016	1.046	0.780	-0.211	0.400	0.000

Beta (13.5–30 Hz)

	dACC	lMFG	rMFG	lIns	rIns	lSMG	rSMG
dACC	0.000	0.555	-0.173	1.947	-0.921	1.419	0.507
lMFG	0.555	0.000	-0.883	-0.511	-0.464	-1.212	-0.410
rMFG	-0.173	-0.883	0.000	0.480	-0.656	-0.063	-0.544
lIns	1.947	-0.511	0.480	0.000	-0.149	0.627	1.084
rIns	-0.921	-0.464	-0.656	-0.149	0.000	-0.550	0.781
lSMG	1.419	-1.212	-0.063	0.627	-0.550	0.000	-1.464
rSMG	0.507	-0.410	-0.544	1.084	0.781	-1.464	0.000

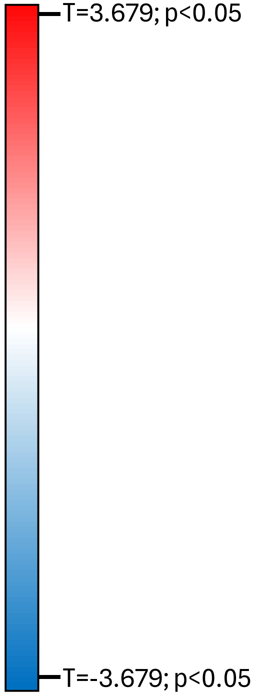

Abbreviations: dACC: dorsal Anterior Cingulate Cortex; lMFG: left Middle Frontal Gyrus; rMFG: right Middle Frontal Gyrus; lIns: left Insula; rIns: right Insula; lSMG: left Supramarginal Gyrus; rSMG: right Supramarginal Gyrus

Figure S5. The complete T-values matrix of functional connectivity results of within-group (BPD) comparison (REC_H vs RS) in all frequency bands (N=24).

REC_H vs RS in BPD

Delta (1-4 Hz)

	dACC	lMFG	rMFG	lIns	rIns	lSMG	rSMG
dACC	0.000	2.331	2.127	1.207	2.417	2.267	1.390
lMFG	2.331	0.000	0.681	2.295	1.721	2.789	2.034
rMFG	2.127	0.681	0.000	1.330	2.714	3.040	1.405
lIns	1.207	2.295	1.330	0.000	1.570	1.741	0.323
rIns	2.417	1.721	2.714	1.570	0.000	1.059	0.843
lSMG	2.267	2.789	3.040	1.741	1.059	0.000	1.415
rSMG	1.390	2.034	1.405	0.323	0.843	1.415	0.000

Theta (4.5-7.5 Hz)

	dACC	lMFG	rMFG	lIns	rIns	lSMG	rSMG
dACC	0.000	2.226	0.731	1.232	2.352	2.435	0.629
lMFG	2.226	0.000	2.163	1.962	2.375	2.557	1.567
rMFG	0.731	2.163	0.000	3.116	3.068	1.716	3.043
lIns	1.232	1.962	3.116	0.000	1.125	2.624	1.028
rIns	2.352	2.375	3.068	1.125	0.000	3.013	2.806
lSMG	2.435	2.557	1.716	2.624	3.013	0.000	1.803
rSMG	0.629	1.567	3.043	1.028	2.806	1.803	0.000

Alpha (8–13 Hz)

	dACC	lMFG	rMFG	lIns	rIns	lSMG	rSMG
dACC	0.000	0.880	2.900	2.729	2.124	1.290	1.304
lMFG	0.880	0.000	1.921	0.893	1.284	0.688	1.419
rMFG	2.900	1.921	0.000	2.518	1.377	3.134	1.981
lIns	2.729	0.893	2.518	0.000	1.119	-0.588	2.014
rIns	2.124	1.284	1.377	1.119	0.000	3.399	0.618
lSMG	1.290	0.688	3.134	-0.588	3.399	0.000	0.416
rSMG	1.304	1.419	1.981	2.014	0.618	0.416	0.000

Beta (13.5–30 Hz)

	dACC	lMFG	rMFG	lIns	rIns	lSMG	rSMG
dACC	0.000	-0.416	0.143	-0.620	0.810	-0.691	-0.054
lMFG	-0.416	0.000	-0.926	-1.620	1.176	-0.886	1.111
rMFG	0.143	-0.926	0.000	0.742	0.029	0.837	0.964
lIns	-0.620	-1.620	0.742	0.000	0.576	-0.341	0.319
rIns	0.810	1.176	0.029	0.576	0.000	-0.203	1.262
lSMG	-0.691	-0.886	0.837	-0.341	-0.203	0.000	1.706
rSMG	-0.054	1.111	0.964	0.319	1.262	1.706	0.000

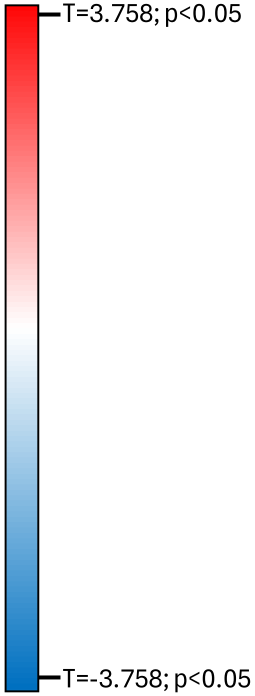

Abbreviations: dACC: dorsal Anterior Cingulate Cortex; lMFG: left Middle Frontal Gyrus; rMFG: right Middle Frontal Gyrus; lIns: left Insula; rIns: right Insula; lSMG: left Supramarginal Gyrus; rSMG: right Supramarginal Gyrus

Figure S6. The complete T-values matrix of functional connectivity results of within-group (BPD) comparison (REC_F vs RS) in all frequency bands (N=24).

REC_F vs RS in BPD

Delta (1-4 Hz)

	dACC	lMFG	rMFG	lIns	rIns	lSMG	rSMG
dACC	0.000	**3.924**	2.064	2.614	2.558	2.085	2.570
lMFG	**3.924**	0.000	0.242	2.370	2.854	2.088	2.622
rMFG	2.064	0.242	0.000	1.885	2.217	2.918	2.735
lIns	2.614	2.370	1.885	0.000	0.932	2.645	0.652
rIns	2.558	2.854	2.217	0.932	0.000	1.544	2.227
lSMG	2.085	2.088	2.918	2.645	1.544	0.000	2.282
rSMG	2.570	2.622	2.735	0.652	2.227	2.282	0.000

Theta (4.5-7.5 Hz)

	dACC	lMFG	rMFG	lIns	rIns	lSMG	rSMG
dACC	0.000	-0.213	1.584	0.755	1.712	0.346	1.025
lMFG	-0.213	0.000	1.666	1.176	2.282	1.497	1.994
rMFG	1.584	1.666	0.000	2.880	2.120	2.875	1.776
lIns	0.755	1.176	2.880	0.000	2.243	1.790	1.408
rIns	1.712	2.282	2.120	2.243	0.000	1.348	0.792
lSMG	0.346	1.497	2.875	1.790	1.348	0.000	0.727
rSMG	1.025	1.994	1.776	1.408	0.792	0.727	0.000

Alpha (8–13 Hz)

	dACC	lMFG	rMFG	lIns	rIns	lSMG	rSMG
dACC	0.000	0.344	1.549	1.380	2.020	-0.196	1.492
lMFG	0.344	0.000	1.150	1.593	1.547	0.136	1.835
rMFG	1.549	1.150	0.000	1.787	0.970	0.457	2.390
lIns	1.380	1.593	1.787	0.000	0.317	-0.453	1.507
rIns	2.020	1.547	0.970	0.317	0.000	1.049	1.185
lSMG	-0.196	0.136	0.457	-0.453	1.049	0.000	-0.383
rSMG	1.492	1.835	2.390	1.507	1.185	-0.383	0.000

Beta (13.5–30 Hz)

	dACC	lMFG	rMFG	lIns	rIns	lSMG	rSMG
dACC	0.000	-2.713	-1.046	-0.972	-2.524	-0.863	0.004
lMFG	-2.713	0.000	-1.390	-1.658	-1.108	-2.271	0.080
rMFG	-1.046	-1.390	0.000	-0.029	-1.261	-0.177	-0.336
lIns	-0.972	-1.658	-0.029	0.000	-0.893	-1.553	0.022
rIns	-2.524	-1.108	-1.261	-0.893	0.000	-1.372	0.522
lSMG	-0.863	-2.271	-0.177	-1.553	-1.372	0.000	-1.000
rSMG	0.004	0.080	-0.336	0.022	0.522	-1.000	0.000

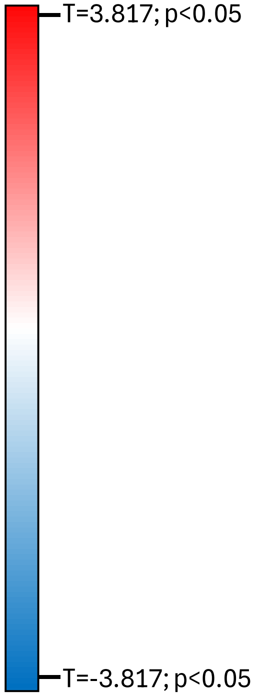

Abbreviations: dACC: dorsal Anterior Cingulate Cortex; lMFG: left Middle Frontal Gyrus; rMFG: right Middle Frontal Gyrus; lIns: left Insula; rIns: right Insula; lSMG: left Supramarginal Gyrus; rSMG: right Supramarginal Gyrus

Figure S7. The complete T-values matrix of functional connectivity results of within-group (BPD) comparison (REC_F vs REC_H) in all frequency bands (N=24).

REC_F vs REC_H in BPD

Delta (1-4 Hz)

	dACC	lMFG	rMFG	lIns	rIns	lSMG	rSMG
dACC	0.000	0.650	0.449	1.234	0.084	0.984	0.810
lMFG	0.650	0.000	-0.360	-0.233	0.721	-0.588	0.108
rMFG	0.449	-0.360	0.000	-0.125	-0.554	-0.231	1.426
lIns	1.234	-0.233	-0.125	0.000	-0.656	0.534	0.085
rIns	0.084	0.721	-0.554	-0.656	0.000	0.416	1.829
lSMG	0.984	-0.588	-0.231	0.534	0.416	0.000	0.595
rSMG	0.810	0.108	1.426	0.085	1.829	0.595	0.000

Theta (4.5-7.5 Hz)

	dACC	lMFG	rMFG	lIns	rIns	lSMG	rSMG
dACC	0.000	-2.125	0.786	-0.802	-0.901	-1.705	0.359
lMFG	-2.125	0.000	-0.911	-0.924	-0.158	-0.928	-0.124
rMFG	0.786	-0.911	0.000	0.047	-1.244	1.309	-1.325
lIns	-0.802	-0.924	0.047	0.000	0.817	-0.100	0.014
rIns	-0.901	-0.158	-1.244	0.817	0.000	-1.333	-1.572
lSMG	-1.705	-0.928	1.309	-0.100	-1.333	0.000	-1.312
rSMG	0.359	-0.124	-1.325	0.014	-1.572	-1.312	0.000

Alpha (8–13 Hz)

	dACC	lMFG	rMFG	lIns	rIns	lSMG	rSMG
dACC	0.000	-0.366	-0.913	-0.304	-0.466	-1.074	0.746
lMFG	-0.366	0.000	-0.311	0.718	-0.051	-0.460	0.634
rMFG	-0.913	-0.311	0.000	-0.716	-0.415	-2.710	0.247
lIns	-0.304	0.718	-0.716	0.000	-0.655	0.007	0.307
rIns	-0.466	-0.051	-0.415	-0.655	0.000	-1.556	0.344
lSMG	-1.074	-0.460	-2.710	0.007	-1.556	0.000	-0.879
rSMG	0.746	0.634	0.247	0.307	0.344	-0.879	0.000

Beta (13.5–30 Hz)

	dACC	lMFG	rMFG	lIns	rIns	lSMG	rSMG
dACC	0.000	-2.127	-1.055	-0.517	-2.079	-0.290	0.063
lMFG	-2.127	0.000	-0.716	0.118	-2.256	-1.289	-1.046
rMFG	-1.055	-0.716	0.000	-0.754	-1.437	-0.957	-1.201
lIns	-0.517	0.118	-0.754	0.000	-1.524	-1.184	-0.251
rIns	-2.079	-2.256	-1.437	-1.524	0.000	-1.495	-0.701
lSMG	-0.290	-1.289	-0.957	-1.184	-1.495	0.000	-2.152
rSMG	0.063	-1.046	-1.201	-0.251	-0.701	-2.152	0.000

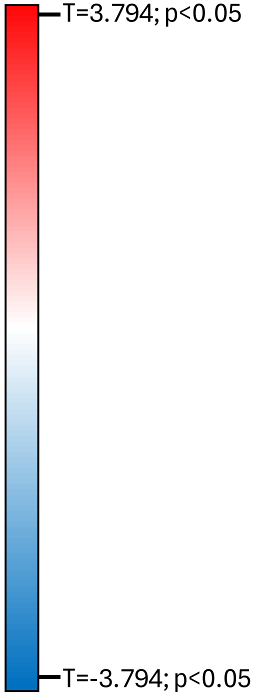

Abbreviations: dACC: dorsal Anterior Cingulate Cortex; lMFG: left Middle Frontal Gyrus; rMFG: right Middle Frontal Gyrus; lIns: left Insula; rIns: right Insula; lSMG: left Supramarginal Gyrus; rSMG: right Supramarginal Gyrus

Figure S8. The complete T-values matrix of functional connectivity results of within-group (HC) comparison (REC_H vs RS) in all frequency bands (N=15).

REC_H vs RS in HC

Delta (1-4 Hz)

	dACC	lMFG	rMFG	lIns	rIns	lSMG	rSMG
dACC	0.000	0.017	0.866	0.476	-0.284	0.677	0.787
lMFG	0.017	0.000	0.497	0.362	0.018	0.989	0.456
rMFG	0.866	0.497	0.000	0.862	0.014	0.651	0.697
lIns	0.476	0.362	0.862	0.000	0.388	0.504	1.688
rIns	-0.284	0.018	0.014	0.388	0.000	0.629	0.472
lSMG	0.677	0.989	0.651	0.504	0.629	0.000	2.075
rSMG	0.787	0.456	0.697	1.688	0.472	2.075	0.000

Theta (4.5-7.5 Hz)

	dACC	lMFG	rMFG	lIns	rIns	lSMG	rSMG
dACC	0.000	-0.778	0.315	-2.054	-0.930	0.077	0.244
lMFG	-0.778	0.000	0.919	-0.476	0.110	-1.166	0.938
rMFG	0.315	0.919	0.000	-0.734	-0.680	-0.364	0.069
lIns	-2.054	-0.476	-0.734	0.000	0.471	-0.418	-0.233
rIns	-0.930	0.110	-0.680	0.471	0.000	0.073	0.023
lSMG	0.077	-1.166	-0.364	-0.418	0.073	0.000	-0.104
rSMG	0.244	0.938	0.069	-0.233	0.023	-0.104	0.000

Alpha (8–13 Hz)

	dACC	lMFG	rMFG	lIns	rIns	lSMG	rSMG
dACC	0.000	-0.460	-0.562	0.567	-0.026	0.400	0.768
lMFG	-0.460	0.000	0.575	0.825	1.979	0.513	-0.023
rMFG	-0.562	0.575	0.000	0.889	0.136	-1.586	-1.303
lIns	0.567	0.825	0.889	0.000	2.368	-0.167	0.037
rIns	-0.026	1.979	0.136	2.368	0.000	-1.491	-1.730
lSMG	0.400	0.513	-1.586	-0.167	-1.491	0.000	-0.143
rSMG	0.768	-0.023	-1.303	0.037	-1.730	-0.143	0.000

Beta (13.5–30 Hz)

	dACC	lMFG	rMFG	lIns	rIns	lSMG	rSMG
dACC	0.000	-0.160	0.324	-1.134	-0.835	-0.405	0.922
lMFG	-0.160	0.000	0.178	0.847	0.432	2.052	0.537
rMFG	0.324	0.178	0.000	0.448	-0.073	0.605	1.375
lIns	-1.134	0.847	0.448	0.000	0.405	0.086	0.585
rIns	-0.835	0.432	-0.073	0.405	0.000	0.002	-0.255
lSMG	-0.405	2.052	0.605	0.086	0.002	0.000	1.700
rSMG	0.922	0.537	1.375	0.585	-0.255	1.700	0.000

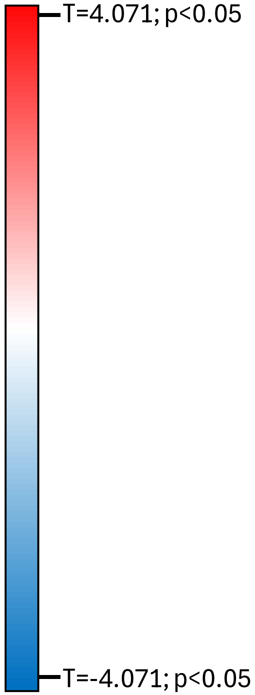

Abbreviations: dACC: dorsal Anterior Cingulate Cortex; lMFG: left Middle Frontal Gyrus; rMFG: right Middle Frontal Gyrus; lIns: left Insula; rIns: right Insula; lSMG: left Supramarginal Gyrus; rSMG: right Supramarginal Gyrus

Figure S9. The complete T-values matrix of functional connectivity results of within-group (HC) comparison (REC_F vs RS) in all frequency bands (N=15).

REC_F vs RS in HC

Delta (1-4 Hz)

	dACC	lMFG	rMFG	lIns	rIns	lSMG	rSMG
dACC	0.000	-0.702	0.765	0.526	-0.681	-0.275	-0.156
lMFG	-0.702	0.000	-0.201	0.971	0.356	1.892	0.133
rMFG	0.765	-0.201	0.000	1.295	0.050	0.289	1.674
lIns	0.526	0.971	1.295	0.000	1.538	-0.410	1.720
rIns	-0.681	0.356	0.050	1.538	0.000	0.018	0.218
lSMG	-0.275	1.892	0.289	-0.410	0.018	0.000	1.654
rSMG	-0.156	0.133	1.674	1.720	0.218	1.654	0.000

Theta (4.5-7.5 Hz)

	dACC	lMFG	rMFG	lIns	rIns	lSMG	rSMG
dACC	0.000	-0.965	-0.006	-2.360	-1.189	-1.674	-0.081
lMFG	-0.965	0.000	0.193	0.686	0.068	-2.653	-1.312
rMFG	-0.006	0.193	0.000	-0.687	1.069	-1.176	-1.414
lIns	-2.360	0.686	-0.687	0.000	-0.506	-1.312	-0.978
rIns	-1.189	0.068	1.069	-0.506	0.000	-2.079	-1.130
lSMG	-1.674	-2.653	-1.176	-1.312	-2.079	0.000	-1.028
rSMG	-0.081	-1.312	-1.414	-0.978	-1.130	-1.028	0.000

Alpha (8–13 Hz)

	dACC	lMFG	rMFG	lIns	rIns	lSMG	rSMG
dACC	0.000	1.415	1.032	1.996	1.117	0.798	1.907
lMFG	1.415	0.000	0.590	1.080	1.451	0.287	0.424
rMFG	1.032	0.590	0.000	1.598	1.014	0.561	1.064
lIns	1.996	1.080	1.598	0.000	1.745	-1.251	1.056
rIns	1.117	1.451	1.014	1.745	0.000	-1.466	0.096
lSMG	0.798	0.287	0.561	-1.251	-1.466	0.000	-2.441
rSMG	1.907	0.424	1.064	1.056	0.096	-2.441	0.000

Beta (13.5–30 Hz)

	dACC	lMFG	rMFG	lIns	rIns	lSMG	rSMG
dACC	0.000	-0.043	1.903	-1.875	-0.246	-0.695	1.431
lMFG	-0.043	0.000	-0.505	-0.078	-0.255	0.932	-0.080
rMFG	1.903	-0.505	0.000	-0.829	-0.428	-0.587	1.898
lIns	-1.875	-0.078	-0.829	0.000	-0.106	-1.102	-1.031
rIns	-0.246	-0.255	-0.428	-0.106	0.000	-0.237	-0.204
lSMG	-0.695	0.932	-0.587	-1.102	-0.237	0.000	1.146
rSMG	1.431	-0.080	1.898	-1.031	-0.204	1.146	0.000

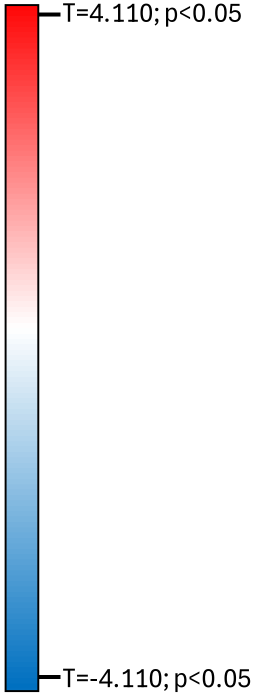

Abbreviations: dACC: dorsal Anterior Cingulate Cortex; lMFG: left Middle Frontal Gyrus; rMFG: right Middle Frontal Gyrus; lIns: left Insula; rIns: right Insula; lSMG: left Supramarginal Gyrus; rSMG: right Supramarginal Gyrus

Figure S10. The complete T-values matrix of functional connectivity results of within-group (HC) comparison (REC_F vs REC_H) in all frequency bands (N=15).

REC_F vs REC_H in HC

Delta (1-4 Hz)

	dACC	lMFG	rMFG	lIns	rIns	lSMG	rSMG
dACC	0.000	-1.369	-0.405	0.106	-0.659	-1.076	-1.775
lMFG	-1.369	0.000	-1.398	0.585	0.495	0.351	-0.608
rMFG	-0.405	-1.398	0.000	0.541	0.054	-0.449	1.848
lIns	0.106	0.585	0.541	0.000	1.158	-0.767	0.614
rIns	-0.659	0.495	0.054	1.158	0.000	-0.482	-0.367
lSMG	-1.076	0.351	-0.449	-0.767	-0.482	0.000	-0.200
rSMG	-1.775	-0.608	1.848	0.614	-0.367	-0.200	0.000

Theta (4.5-7.5 Hz)

	dACC	lMFG	rMFG	lIns	rIns	lSMG	rSMG
dACC	0.000	-0.560	-0.276	0.222	0.085	-1.083	-0.536
lMFG	-0.560	0.000	-1.157	1.182	-0.072	-1.091	-1.923
rMFG	-0.276	-1.157	0.000	-0.149	2.889	-0.986	-1.258
lIns	0.222	1.182	-0.149	0.000	-1.208	-0.655	-0.741
rIns	0.085	-0.072	2.889	-1.208	0.000	-1.662	-1.668
lSMG	-1.083	-1.091	-0.986	-0.655	-1.662	0.000	-1.695
rSMG	-0.536	-1.923	-1.258	-0.741	-1.668	-1.695	0.000

Alpha (8–13 Hz)

	dACC	lMFG	rMFG	lIns	rIns	lSMG	rSMG
dACC	0.000	1.937	1.817	1.485	1.821	0.342	0.544
lMFG	1.937	0.000	-0.302	0.608	-0.713	-0.286	0.353
rMFG	1.817	-0.302	0.000	0.989	0.881	2.317	2.328
lIns	1.485	0.608	0.989	0.000	-0.145	-0.958	0.575
rIns	1.821	-0.713	0.881	-0.145	0.000	0.407	1.876
lSMG	0.342	-0.286	2.317	-0.958	0.407	0.000	-1.279
rSMG	0.544	0.353	2.328	0.575	1.876	-1.279	0.000

Beta (13.5–30 Hz)

	dACC	lMFG	rMFG	lIns	rIns	lSMG	rSMG
dACC	0.000	0.114	0.429	-1.355	0.486	-0.665	0.179
lMFG	0.114	0.000	-0.734	-1.078	-0.817	-0.381	-0.612
rMFG	0.429	-0.734	0.000	-0.860	-0.254	-1.184	-0.674
lIns	-1.355	-1.078	-0.860	0.000	-0.487	-2.075	-2.194
rIns	0.486	-0.817	-0.254	-0.487	0.000	-0.210	0.133
lSMG	-0.665	-0.381	-1.184	-2.075	-0.210	0.000	-0.887
rSMG	0.179	-0.612	-0.674	-2.194	0.133	-0.887	0.000

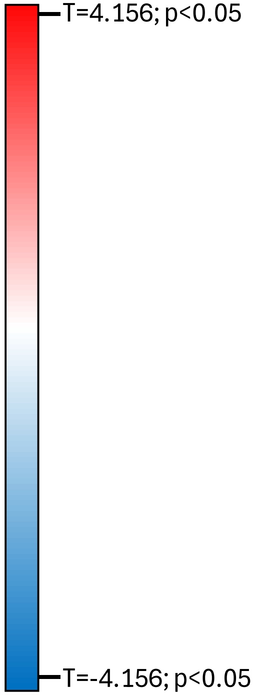

Abbreviations: dACC: dorsal Anterior Cingulate Cortex; lMFG: left Middle Frontal Gyrus; rMFG: right Middle Frontal Gyrus; lIns: left Insula; rIns: right Insula; lSMG: left Supramarginal Gyrus; rSMG: right Supramarginal Gyrus

References

First, M.B.; Williams, J.B.W.; Karg, R.S.; Spitzer, R.L. *User's guide for the scid-5-pd (structured clinical interview for dsm-5 personality)*. American Psychiatric Association Publishing: Washington, DC 2016; p 112

Jørgensen, M.S.; Møller, L.; Bo, S.; Kongerslev, M.; Hastrup, L.H.; Chanen, A.; Storebø, O.J.; Poulsen, S.; Beck, E.; Simonsen, E. The course of borderline personality disorder from adolescence to early adulthood: A 5-year follow-up study. *Compr. Psychiatry* **2024**, *132*, 152478.

Fossati, A.; Borroni, S. *Scid-5-pd - intervista clinica strutturata per i disturbi di personalità del dsm-5*. Raffaello Cortina: Milano, 2017.

Kaufman, J.; Birmaher, B.; Brent, D.; Rao, U.; Flynn, C.; Moreci, P.; Williamson, D.; Ryan, N. Schedule for affective disorders and schizophrenia for school-age children-present and lifetime version (k-sads-pl): Initial reliability and validity data. *J. Am. Acad. Child. Adolesc. Psychiatry* **1997**, *36*, 980-988.

Kaufman, J.; Birmaher, B.; Brent, D.A.; Ryan, N.D.; Rao, U. *K-sads-pl dsm-5 - intervista diagnostica per la valutazione dei disturbi psicopatologici in bambini e adolescenti*. Edizioni Centro Studi Erikson: Trento, 2019.

Gratz, K.L.; Roemer, L. Multidimensional assessment of emotion regulation and dysregulation: Development, factor structure, and initial validation of the difficulties in emotion regulation scale. *J. Psychopathol. Behav. Assess.* **2004**, *26*, 41-54.

Weinberg, A.; Klonsky, E.D. Measurement of emotion dysregulation in adolescents. *Psychol. Assess.* **2009**, *21*, 616-621.

Ibraheim, M.; Kalpakci, A.; Sharp, C. The specificity of emotion dysregulation in adolescents with borderline personality disorder: Comparison with psychiatric and healthy controls. *Borderline Personal. Disord. Emot. Dysregul.* **2017**, *4*, 1.

Harrison, A.; Sullivan, S.; Tchanturia, K.; Treasure, J. Emotional functioning in eating disorders: Attentional bias, emotion recognition and emotion regulation. *Psychol. Assess.* **2010**, *40*, 1887-1897.

Sighinolfi, C.; Norcini Pala, A.; Chiri, L.R.; Marchetti, I.; Sica, C. Difficulties in emotion regulation scale (ders): The italian translation and adaptation. *Psicoter. Cogn. Comport.* **2010**, *16*, 141-170.

Patton, J.H.; Stanford, M.S.; Barratt, E.S. Factor structure of the barratt impulsiveness scale. *J. Clin. Psychol.* **1995**, *51*, 768-774.

Stanford, M.S.; Mathias, C.W.; Dougherty, D.M.; Lake, S.L.; Anderson, N.E.; Patton, J.H. Fifty years of the barratt impulsiveness scale: An update and review. *Pers. Indiv. Differ.* **2009**, *47*, 385-395.

Vasconcelos, A.; Malloy-Diniz, L.; Correa, H. Systematic review of psychometric proprieties of barrattimpulsiveness scale version 11 (bis-11). *Clin. Neuropsychiatry* **2012**, *9*, 61-74.

Fossati, A.; Di Ceglie, A.; Acquarini, E.; Barratt, E.S. Psychometric properties of an italian version of the barratt impulsiveness scale-11 (bis-11) in nonclinical subjects. *J. Clin. Psychol.* **2001**, *57*, 815-828.

Armstrong, J.G.; Putnam, F.W.; Carlson, E.B.; Libero, D.Z.; Smith, S.R. Development and validation of a measure of adolescent dissociation: The adolescent dissociative experiences scale. *J. Nerv. Ment. Dis.* **1997**, *185*, 491-497.

De Pasquale, C.; Sciacca, F.; Hichy, Z. Validation of the italian version of the dissociative experience scale for adolescents and young adults. *Ann. Gen. Psychiatry* **2016**, *15*, 31.

Bernstein, D.P.; Stein, J.A.; Newcomb, M.D.; Walker, E.; Pogge, D.; Ahluvalia, T.; Stokes, J.; Handelsman, L.; Medrano, M.; Desmond, D.*, et al.* Development and validation of a brief

screening version of the childhood trauma questionnaire. *Child Abuse Negl.* **2003**, *27*, 169-190.
Hagborg, J.M.; Kalin, T.; Gerdner, A. The childhood trauma questionnaire—short form (ctq-sf) used with adolescents – methodological report from clinical and community samples. *J. Child Adolesc. Trauma* **2022**, *15*, 1199-1213.
Sacchi, C.; Vieno, A.; Simonelli, A. Italian validation of the childhood trauma questionnaire—short form on a college group. *Psychol. Trauma* **2018**, *10*, 563.
